# Which technical difficulty score can best predict postoperative outcomes after minimally invasive liver resections?

**DOI:** 10.1007/s00423-025-03612-z

**Published:** 2025-02-21

**Authors:** Louisa Bolm, Martina Nebbia, Onofrio Catalano, Gabriella Lionetto, Johanna von Bresinsky, Jannis Duhn, Shahrzad Arya, Marco Ventin, Julia Straesser, Cristina R. Ferrone

**Affiliations:** 1 Department of Gastrointestinal Surgery, Massachusetts General Hospital, Harvard Medical School, Boston, MA USA; 2 Department of Radiology, Massachusetts General Hospital, Harvard Medical School, Boston, MA USA; 3 Department of Surgery, University Medical Center Schleswig-Holstein, Campus Lübeck, Germany; 4 Department of Surgery, Unit of Pancreatic Surgery, Humanitas Research Hospital, Milan, Italy; 5 Department of Surgery, Unit of pancreatic Surgery, Verona University, Verona, Italy; 6 Department of Surgery, Cedars-Sinai Medical Center, 8700 Beverly Boulevard, North Tower STE, Los Angeles, 8215 CA USA

**Keywords:** Liver surgery, Laparoscopic liver resection, Technical difficulty

## Abstract

**Background:**

To assess technical difficulty scores for laparoscopic liver resections (LLR) in a large well-characterized cohort of low to high difficulty LLR.

**Methods:**

Patients undergoing LLR and open liver resection (OLS) (2007–2022) at Massachusetts General Hospital were included. Patients were classified according to the technical difficulty scores Ban difficulty score, IWATE criteria, Hasegawa score, IMM score, and Southhampton score (SHH) and calibration of these scores in predicting postoperative outcome parameters was assessed.

**Results:**

301 patients underwent LLR. Median age was 59 years and 58.5% of the patients were female. Median lesion size was 42.2 mm, median operative time was 197.7 min, and median estimated blood loss was 400.5 ml. According to the different scoring systems, 18.9% (SHH) to 52.2% (IWATE) of the LLR were high difficulty. Overall intraoperative events according to the modified Satava classification grade II (6.6%) and grade III (2.7%) were low as was postoperative 90 days major morbidity (5.3%) and mortality (1.0%). The respective scores’ calibration for predicting non-textbook outcomes, intraoperative events, operative time, major postoperative morbidity, blood transfusion rates, and length of hospital stay was moderate to good for the respective scores and best for the IWATE criteria.

**Discussion:**

Even high technical difficulty LLR can be performed with low postoperative morbidity and mortality rates. The scores evaluated performed well in predicting major liver surgery outome parameters. Among the different difficulty scoring systems, the IWATE criteria performed best.

## Introduction

Minimally Invasive techniques have been rapidly emerging in gastrointestinal surgery over the past three decades [1–3]. While minimally-invasive approaches are already established as the preferred option in several fields of oncological abdominal surgery, hepato-pancreatico-biliary (HPB) surgery resections around the world are still mainly performed as open operations [4]. A considerable proportion of HPB operations are highly complex procedures that make a laparoscopic approach challenging and difficult to master [5, 6].

Liver resections cover a wide range of difficulty from small superficial atypical resections to major hepatectomies [7, 8]. While laparoscopic surgery is becoming a routine approach for smaller easily accessible liver lesions, it remains rare for major liver resections [9, 10]. Preoperatively assessing the level of difficulty in order to determine if liver resections can be attempted safely with laparoscopy given the surgeon’s status of laparoscopic training becomes essential [11, 12]. To help guide surgeons as they climb the learning curve several classification systems to outline the technical difficulty of different hepatic resections have been established [11, 13, 14]. Ban et al. scaled difficulty based on perioperative parameters, while more current scoring systems added further patient-related parameters in order to differentiate low to intermediate to high difficulty cases [11, 15]. The Ban score published in 2014 describes anatomical and peripheral left LLR as easier to operations than large, central or posterolateral lesions that are difficult to access with a laparoscopic approach [11]. The Ban scoring system was further refined into the IWATE criteria which differentiates four instead of three difficulty levels adding an expert level [13]. The IWATE criteria are more complex and consider tumor location, extent of a hepatic resection, tumor size, proximity to a major blood vessel, and Child–Pugh liver function. Following these two scoring systems, further technical difficulty classifications were developed based on different international cohorts such as the Japanese Hasegawa score, the French IMM score, and the European Southhampton score [14, 16, 17].

The main purpose of these difficulty scores is to help liver surgeons assess the technical complexity of planned procedures upfront in order to determine if a procedure can be safely performed laparoscopically based on their level of expertise and experience.

We aim to compare the different well known difficulty scoring systems in their ability to predict peri operative outcomes after minor and major laparoscopic liver resections. By limiting the cases to a single surgeon’s experience we will compare the different scoring systems ability to predict the perioperative outcomes in terms of conversion to open, operative time, blood loss, length of stay, major morbidity and mortality.

## Materials and methods

### Study protocol and data collection

Approval for the study was obtained from the Massachusetts General Hospital Institutional Review Board (IRB) (#2020P002635). A retrospective study was conducted evaluating all patients undergoing laparoscopic liver resections (LLR) performed by one expert liver surgeon (CRF) between 2007 and 2022. Only patients with first-time liver resection were included, resections for recurrence or secondary liver surgery were excluded. Patient with singular or multiple lesions resected in one procedure were included, those resected in consecutive multiple step procedures were excluded from the study. All study parameters were derived from a prospectively maintained institutional database. Baseline, surgical, histopathological, surgical and post-operative parameters were collected.

## Baseline, intraoperative, histopathological and postoperative parameters

Baseline parameters included age, gender, American Society of Anesthesiologists (ASA) score, body mass index (BMI), obesity, preoperative serum albumin in mg/dl, and preoperative serum bilirubin in mg/dl. Surgical parameters involved surgical procedure, minor (atypical resection, segmentectomy or bisegmentectomy) and major resections (trisegmentectomy, left and right hepatectomy), operation time in minutes, estimated blood loss in ml, rate of unplanned conversion to open resection and lesion size in mm. Intraoperative complications were assessed based on the modified Satava criteria as grade I to III. Grade 1 involves intraoperative incidents including those with no change to the operative approach and without further consequences for the patient such as higher blood loss; grade 2 incidents have further consequences for the patient such as conversion to open surgery, whereas grade 3 events are those with significant consequences for the patient [18]. Histopathologic parameters collected involved histology of the lesion resected: Primary hepatobiliary cancer, Hepatocellular carcinoma (HCC), hilar cholangiocarcinoma (HCCC), intrahepatic cholangiocarcinoma (IHCCC), gallbladder cancer (GBC)), metastatic lesions (colorectal liver metastases versus others) and benign lesions. Postoperative parameters included postoperative complications according to the Clavien Dindo Classification [19], blood transfusions within 72 h of surgery, total number of units of erythrocyte concentrates transfused, bile leak, superficial surgical site infection, deep incisional surgical site infection, postoperative pneumonia, postoperative stroke, postoperative renal insufficiency, postoperative cardiac event, postoperative sepsis and length of hospital stay. Furthermore, overall hospital stay in days, readmission within 30 days and 90-days postoperative mortality were evaluated. Textbook outcomes in liver surgery were evaluated according to Görgec et al. [20]. Textbook outcome in laparoscopic liver surgery was defined as the absence of intraoperative incidents of grade 2 or higher, postoperative bile leak grade B or C, severe postoperative complications, readmission within 30 days after discharge, in-hospital mortality, and the presence of R0 resection margin.

## Technical difficulty scores

Technical difficulty of liver resection was evaluated with different established difficulty scoring systems. The Ban difficulty scoring system is based on the surgeon’s assessment of difficulty and attributes an index of 1–10 with the following categories: 1–3 points is considered low difficulty, while 4–6 equals intermediate and 7–10 means high difficulty [11]. The IWATE Criteria comprise six variables with a total score ranging from 0 to 12 points [13]. These variables included: tumor location (1–5 points), extent of a hepatic resection (0–4 points), tumor size ≥ 3 or < 3 cm (0–1 points), proximity to a major blood vessel (0–1 points), Child–Pugh liver function (0–1 points), and HALS/hybrid resection (–1–0 points). The 0–12 difficulty index was further subdivided into four difficulty levels: low (0–3), intermediate (4–6), advanced (7–9), and expert (10–12). The Hasegawa score considers four predictive factors and is based on the weighted contribution of each factor: extent of resection (scored 0, 2, or 3); location of tumor (scored 0, 1, or 2); obesity (scored 0 or 1); and platelet count (scored 0 or 1) [16]. The scores are summed to classify surgical difficulty into three levels: low (total score ≤ 1); medium (total score 2–3); and high (total score ≥ 4). The Institut Mutualiste Montsouris Classification system (IMM) involves three different grades of difficulty: Grade 1 (low difficulty level) includes wedge resection and left lateral sectionectomy; Grade 2 (intermediate difficulty level) includes anterolateral segmentectomy (segments 2, 3, 4b, 5, or 6) and left hepatectomy; and Grade 3 (high difficulty level) includes posterosuperior segmentectomy (segments 1, 4a, 7, or 8), right posterior sectionectomy, right hepatectomy, extended right hepatectomy, central hepatectomy, and extended left hepatectomy [17]. The Southhampton difficulty scoring system (SHH) classifies patients into 4 difficulty levels: low (≤ 2), moderate (3–5), high (6–9) and extremely high (10–15) [14]. Neoadjuvant chemotherapy, lesion type and size, classification of resection and previous open liver resection are the determinants of difficulty.

### Statistical analysis

For statistical analysis, IBM SPSS Statistics for Windows, Version 25.0 was used. Continuous and categorical variables were expressed as median/standard error and absolute/relative frequencies. Statistical testing was performed by Chi-squared test for categorical variables and Mann Whitney U test for metric variables. Calibration of difficulty scores was performed using a Spearman rank correlation coefficient, a nonparametric measure of rank correlation (statistical dependence between the rankings of variables). It assesses how well the relationship between two variables can be described using a monotonic function. Higher Spearman Rho (SR) values indicate a higher level of correlation. Calibration was assessed as good if the test reach significance (*p* < 0.05), moderate of there was a trend for correlation (*p* < 0.10), and poor if there was no significant test result or trend detected (*p* > 0.10). The confidence intervals described are 95% confidence intervals. The significance level was set to *p* < 0.05 (two-sided).

## Results

A total of 301 patients had laparoscopic liver resections (LLR) and were included in the study. Median age was 59.2 years and 58.5% LLR patients were female (*p* = 0.4) (Table 1). Mean platelet count was 234.1/ml, mean INR was 1.1 and mean Child-Pugh score was 5.1. 244 (81.0%) patients had minor resections while 57 (19.0%) patients had major resections. 81.4% of patients had a singular lesion resected, the remaining patients had two to up to eight lesions that were all resected in one procedure. Regarding histopathological parameters, patients with LLR had primary hepatobiliary cancers in 29.2%, metastatic cancer in 29.6% and benign lesions in 41.3% of the patients (Table 2). Intraoperative complications according to the modified Satava classification occurred in 20 (6.6%) patients as grade II and in 8 (2.7%) patients as grade III. Unplanned conversion to open surgery occurred in 18 (6.0%) patients. Textbook outcomes for laparoscopic surgery were obtained in 235 (78.1%) patients. Failure to achieve textbook outcomes was mostly related to readmission within 30 days (8.4%). Clavien Dindo Classification complications up to grade IIIa occurred in 25 (8.3%) patients while 16 (5.3%) patients had major postoperative morbidity grade IIIb-IVb. Details of postoperative complications are depicted in Table 3.

**Table 1:** Baseline and surgical parameters in LLR

Baseline and surgical parameters	
	LLR
total n	301
**Parameter**	n(%)/mean (STD)
**Age**	59.2 (15.5)
**Gender**	
Female	176 (58.5)
Male	125 (41.5)
**ASA Score**	
III-IV	139 (46.5)
**Obesity**	94 (31.4)
**BMI (kg/m2)**	28.3 (6.3)
**BMI > 30 kg/m2**	94 (32.1)
**Preoperative serum albumin (mg/dL)**	4.3 (0.5)
**Preoperative serum bilirubin (mg/dL)**	0.5 (0.01)
**Platelet count (/ml)**	234.1 (10.4)
**INR**	1.1 (0.1)
**Child Pugh Score**	5.1 (0.9)
**Surgical Parameters**	
** Minor resections**	244 (81.0)
Atypical resection	94 (31.2)
Segmentectomy	49 (16.3)
Bisegmentectomy	101 (33.6)
** Major resections**	57 (18.9)
Trisegmentectomy	10 (3.3)
Right hepatectomy	30 (10.0)
Left hepatectomy	17 (5.6)
**Operative time** (min)	197.7 (112.6)
**Estimated blood loss (mL)**	400.5 (35.6)
**Lesion size (mm)**	42.2 (1.3)
**Singular lesion**	245 (81.4)

*LLR *Laparoscopic liver resection, *ASA *American Society of Anesthesiology, *BMI *Body mass index

**Table 2:** Histopathological parameters

Histopathology	LLR
**Histopathological diagnosis**	*n (%)*
*Total n*	301
**Primary hepatobiliary cancer**	88 (29.2)
Hepatocellular carcinoma	59 (19.6)
Hilar cholangiocarcinoma	0 (0.0)
Intrahepatic cholangiocarcinoma	16 (5.3)
Gallbladder cancer	12 (4.0)
**Metastatic cancer**	89 (29.6)
Colorectal liver metastases	58 (19.3)
Other	31 (10.2)
**Benign lesions**	124 (41.3)

*LLR *Laparoscopic liver resection

**Table 3:** Postoperative morbidity in LLR patients

LLR	
total n	301
**Parameter**	*n *(%)/median (STD)
*Intraoperative Complications*	
Modified Satava classification Grade II	20 (6.6)
Modified Satava classification Grade III	8 (2.7)
Unplanned conversion to open surgery	18 (6.0)
*Postoperative Complications*	
Clavien Dindo Classification 0	260 (86.4)
Clavien Dindo Classification I-IIIa	25 (8.3)
Major morbidity Clavien Dindo Calssification IIIb-IVb	16 (5.3)
Blood transfusion within 72 h of surgery	27 (9.1)
Units of blood transfusion	3.4 (0.4)
Bile leak Grade B/C	4 (1.3)
Superficial surgical site infection	4 (1.3)
Deep incisional surgical site infection	0 (0.0)
Postoperative pneumonia	3 (1.0)
Postoperative renal insufficiency	3 (1.0)
Postoperative stroke	1 (0.3)
Postoperative cardiac event	0 (0.0)
Postoperative sepsis	4 (1.3)
Length of hospital stay in days	3.8 (0.6)
Readmission rate within 30 days	25 (8.4)
90-days postoperative mortality	4 (1.0)
*Textbook outcomes LLR*	
No textbook outcome	66 (21.9)
Textbook outcome	235 (78.1)

### Technical difficulty scores and postoperative morbidity and outcomes

Technical difficulty in LLR was assessed with different scoring systems, results are detailed in Table 4. Major outcome parameters including textbook outcome, modified Satava classification grade II and higher, postoperative blood transfusion within 72 h, units of blood transfused, length of hospital stay, and 90-day postoperative morbidity (Clavien grade *≥* 3) and mortality were compared between the difficulty levels of the described technical difficulty scores in LLR patients. The rate of textbook outcome cases decreased with the level of technical difficulty ranging from 88.0 to 99.0% for low difficulty and from 44.4 to 66.3% for high or expert difficulty cases. In higher difficulty cases, failure to reach textbook outcome was mostly driven by 30-day readmission. Modified Satava classification grade II and higher events increased with the level of difficulty for all scoring systems except for the SHH score. For low difficulty operations modified Satava classification grade II and higher rates ranged from 1.0 to 1.3%, while they reached rates of 2.7–5.6% for high or expert difficulty operations. Operative times increased with the level of technical difficulty ranging from a mean of 65–93 min for low difficulty to a mean of 204 to 264 min for high or expert difficulty. Severe postoperative morbidity increased with technical difficulty, ranging from 0.0 to 2.2%, while they reached rates of 2.3–5.3% for high or expert difficulty except for the Hasegawa score. Postoperative blood transfusion rates within 72 h increased with level of technical difficulty for all scoring systems. For low difficulty operations blood transfusion rates within 72 h ranged from 1.3 to 2.2%, while they reached rates of 2.3–7.0% for high or expert difficulty operations. Units of blood transfused did not differ between difficulty levels for any score. Length of hospital stay further increased with the level of technical difficulty and was accurately differentiated by all scores based on difficulty, ranging from a median of 1.8 to 2.6 days in low difficulty cases to a median of 4.9 to 6.5 days in high or expert difficulty patients. Postoperative mortality at 90 days was not different between difficulty levels most likely due to the low number of events (Table 5).

**Table 4:** Technical difficulty score overview

Scoring systems difficulty levels LLR	
	LLR
	*n (%)/median (range)*
*Total n*	301
**Ban difficulty scoring system**	
Low difficulty	75 (24.9)
Intermediate difficulty	66 (21.9)
High difficulty	160 (53.2)
Median absolute difficulty score points	3 (1–10)
**IWATE criteria**	
Low difficulty	76 (25.2)
Intermediate difficulty	68 (22.6)
Advanced difficulty	39 (13.0)
Expert difficulty	118 (39.2)
Median absolute difficulty score points	7 (1–10)
**Hasegawa score**	
Low difficulty	102 (33.9)
Intermediate difficulty	56 (18.6)
High difficulty	143 (47.5)
Median absolute difficulty score points	3 (1–7)
**IMM score**	
Group I	126 (41.9)
Group II	76 (25.2)
Group III	99 (32.9)
Median absolute difficulty score points	1 (1–3)
**Southhampton difficulty score**	
Low difficulty	142 (47.2)
Moderate difficulty	102 (33.9)
High difficulty	57 (18.9)
Median absolute difficulty score points	3 (1–9)

*LLR *Laparoscopic liver resection

**Table 5:** Morbidity and outcomes in LLR based of technical difficulty scores

	Textbook outcomes		Satava grade II and higher		Operative time		Major morbidity (CDC > IIIa)		Blood transfusion within 72 h		Units of blood transfusion		Length of hospital stay in days		90-days postoperative mortality	
	*n* (%)	*p*-value	*n* (%)	*p*-value	mean (SE)	*p*-value	*n* (%)	*p*-value	*n* (%)	*p*-value	mean (SE)	*p*-value	mean (SE)	*p*-value	*n* (%)	*p*-value
Total *n* 301	Total *n* 236		Total *n* 28				Total *n* 16								Total *n* 4	
**Ban score**																
Low	70 (92.1)		3 (1.0)		72 (52)		0 (0.0)		4 (5.6)		2.3 (0.7)		1.8 (0.4)		0 (0.0)	
Intermediate	60 (90.9)		10 (3.3)		156 (122)		6 (2.0)		2 (3.0)		2.5 (0.8)	0.872	3.1 (0.9)		1 (1.5)	
High	106 (66.3)	**0.002**	15 (5.0)	**0.050**	233 (165)	**0.041**	10 (3.3)	**0.003**	21 (16.7)	**0.032**	3.7 (0.7)	0.686	4.9 (1.1)	**0.001**	3 (4.7)	0.390
**IWATE criteria**																
Low	71 (97.3)		3 (1.0)		79 (48)		1 (0.3)		4 (5.6)		2.3 (0.7)		1.9 (0.5)		0 (0.0)	
Intermediate	63 (92.6)		7 (2.3)		123 (101)		5 (1.7)		2 (2.9)		2.5 (0.8)	0.867	3.4 (1.1)		1 (1.5)	
Advanced	30 (76.9)		10 (3.3)		178 (123)		4 (1.3)		14 (11.9)		3.7 (0.6)	0.287	4.5 (0.8)		1 (0.8)	
Expert	72 (61.0)	**0.001**	8 (2.7)	**0.031**	220 (128)	**0.032**	7 (2.3)	**0.041**	7 (17.9)	**0.057**	3.6 (0.8)	0.940	6.3 (1.3)	**0.015**	2 (5.1)	0.194
**Hasegawa score**																
Low	100 (99.0)		4 (1.3)		65 (32)		7 (2.3)		5 (5.1)		2.4 (0.2)		2.1 (0.5)		1 (1.3)	
Intermediate	45 (80.4)		9 (3.0)		98 (72)		8 (2.7)		2 (3.6)		2.0 (0.0)	0.762	3.6 (0.5)		0 (0.0)	
High	91 (63.6)	**0.004**	17 (5.6)	**0.023**	204 (65)		1 (0.3)	0.287	20 (14.0)	**0.017**	3.75 (0.6)	0.554	5.0 (1.1)	**0.018**	3 (2.1)	0.535
**IMM score**																
Group I	120 (95.2)		4 (1.3)		93 (34)		0 (0.0)		6 (4.9)		2.3 (0.5)		2.4 (0.7)		1 (1.0)	
Group II	65 (85.6)		11 (3.7)		158 (125)		0 (0.0)		3 (3.9)		2.0 (0.7)	0.743	3.7 (1.1)		0 (0.0)	
Group III	51 (51.5)	**0.001**	13 (4.3)	**0.048**	259 (149)	**0.024**	16 (5.3)	**0.021**	18 (18.2)	**0.001**	3.9 (0.9)	0.443	5.6 (0.6)	**0.002**	3 (3.0)	0.225
**SHH**																
Low	125 (88.0)		3 (1.0)		79 (34)		0 (0.0)		6 (4.3)		2.3 (1.5)		2.6 (0.6)		0 (0.0)	
Moderate	87 (85.2)		12 (4.0)		165 (136)		5 (1.7)		10 (9.8)		4.5 (1.3)	0.354	4.0 (0.9)		2 (2.0)	
High	24 (44.4)		13 (4.3)	0.180	264 (142)	**0.045**	11 (3.7)	**0.002**	11 (19.6)	**0.009**	2.9 (1.0)	0.369	6.5 (1.5)	**< 0.001**	2 (3.6)	0.160

*LLR *Laparoscopic liver resection

### Calibration and accuracy of the different difficulty scores in predicting perioperative parameters

As described above, all scoring systems succeeded in differentiating low versus higher rates of postoperative severe complications based on technical difficulty. We further aimed to compare the different scoring systems in their ability to predict different rates of perioperative outcomes and postoperative complications using the Spearman Rho calibration coefficient test. We further aimed to determine the calibration of the scores regarding the respective difficulty levels and their correlation with textbook outcomes. There was a good calibration for the Ban score (SR 0.418, 95% CI 0.289–0.524, *p* = 0.002), the IWATE score (SR 0.486, 95% CI 0.311–0.589, *p* = 0.001), the Hasegawa score (SR 0.427, 95%CI 0.210–0.688, *p* = 0.003), the IMM score (SR 0.398, 95% CI 0.237–0.578, *p* = 0.005) and the SHH score (SR 0.367, 95% CI 0.167–0.403, *p* = 0.007). There was a good calibration for the Ban score (SR 0.427, 95% CI 0.298–0.516, *p* = 0.003), the IWATE score (SR 0.478, 95% CI 0.311–0.587, *p* = 0.001), the Hasegawa score (SR 0.411, 95%CI 0.301–0.499, *p* = 0.004), the IMM score (SR 0.429, 95% CI 0.376–0.501, *p* = 0.004) and the SHH score (SR 0.416, 95% CI 0.376–0.511, *p* = 0.0005) in assessing any postoperative morbidity. We further aimed to determine the calibration of the scores regarding the respective difficulty levels and their correlation with modified Satava classification grade II and higher. There was a good calibration for the Ban score (SR 0.372, 95% CI 0.299–0.428, *p* = 0.003), the IWATE score (SR 0.387, 95% CI 0.303–0.407, *p* = 0.003), the Hasegawa score (SR 0.322, 95%CI 0.280–0.398, *p* = 0.008), the IMM score (SR 0.263, 95% CI 0.211–0.329, *p* = 0.018) and moderate calibration for the SHH score (SR 0.177, 95% CI 0.103–0.267, *p* = 0.068). We further aimed to determine the calibration of the scores regarding the respective difficulty levels and their correlation with different rates of postoperative blood transfusion. There was a poor calibration for the Ban score (SR 0.288, 95% CI 0.182–0.398, *p* = 0.143), the IWATE score (SR 0.254, 95% CI 0.211–0.387, *p* = 0.288), the Hasegawa score (SR 0.124, 95%CI 0.101–0.292, *p* = 0.287), the IMM score (SR 0.145, 95% CI 0.111–0.478, *p* = 0.398) and moderate calibration for the SHH score (SR 0.426, 95% CI 0.356–0.545, *p* = 0.090). Therefore, none of the scores was well-calibrated to measure an increase in blood transfusion rates based on increased level of difficulty. We further aimed to determine the calibration of the respective scores to correlate length of hospital stay to the level of difficulty. There was a good calibration for the Ban score (SR 0.489, 95% CI 0.411–0.567, *p* = 0.001), the IWATE score (SR 0.490, 95% CI 0.410–0.599, *p* = 0.001), the Hasegawa score (SR 0.474, 95%CI 0.399–0.582, *p* = 0.001), the SHH score (SR 0.410, 95% CI 0.344–0.578, *p* = 0.009), but only a moderate calibration for the IMM score (SR 0.220, 95% CI 0.200–0.387, *p* = 0.302). Therefore, all scores except from the IMM score calibrated well for length of hospital stay. We further aimed to determine the scores’ calibration to differentiate durations of operative times based on difficulty level. There was a good calibration for the Ban score (SR 0.324, 95% CI 0.323–0.421, *p* = 0.042), the IWATE score (SR 0.389, 95% CI 0.321–0.452, *p* = 0.032), the IMM score (SR 0.289, 95% CI 0.201–0.306, *p* = 0.029), but poor calibration for the Hasegawa score (SR 0.096, 95%CI 0.068–0.110, *p* = 0.390) and the SHH score (SR 0.011, 95% CI 0.008–0.122, *p* = 0.906). All scores except from the Hasegawa and SHH score calibrated well for operative times.

## Discussion

Laparoscopic abdominal surgery is on the rise even for complex oncological procedures in hepato-pancreatico-biliary surgery [21, 22]. Potential advantages of LLR involve improved short-term outcomes with a decrease in postoperative morbidity, reduced perioperative blood loss, less post-operative pain, and faster return of function [8, 23]. The current study demonstrates that laparoscopic liver resections (LLR) in the hands of an experienced liver surgeon can be performed with low overall morbidity in the context of the current literature at all technical difficulty stages up to expert difficulty [24, 25]. This is the first study to compare all five major difficulty scoring systems. Among the different difficulty scoring systems, the IWATE criteria performed best in differentiating major perioperative outcome parameters.

LLR are complex procedures that often require different approaches than OLR. Therefore, these procedures are best performed at expert hepatobiliary surgical centers and by highly experienced surgeons [20, 26]. Performing LLR requires both experience in OLR, as well as advanced laparoscopic skills [23]. While minor resections such as atypical resections or segmentectomies have become wide-spread, major LLR are still limited to very few tertiary centers internationally [26]. Due to the wide range of technical difficulty in LLR, several groups have established different difficulty scoring systems [14, 17]. The main purpose of these difficulty scores is to help liver surgeons assess the technical complexity of planned procedures upfront in order to determine if a procedure can be safely performed laparoscopically based on their level of expertise and experience. Our study demonstrates that all of these scoring systems accurately categorize operations in different cohorts of difficulty. The rates of postoperative morbidity as well as transfusions and length of hospital stay increased with level of difficulty for all technical difficulty scores assessed. Regarding accuracy and calibration, the IWATE criteria performed best and best differentiated all major perioperative outcome parameters. The IWATE criteria classifies patients into four different categories, and is the most complex scoring system incorporating aspects of lesion accessibility, extent of resection, lesion size, vessel proximity and patient factors such as Child Pugh score. It can therefore be assumed that an ideal difficulty score takes into consideration a multitude of not only anatomical and technical aspects, but also patient-related factors. In line with our results, Barron et al. validated the IWATE criteria in an independent cohort and found them to accurately distinguish between patients at low versus high risk of perioperative complications [13]. Ivanecz et al. also evaluated the IWATE criteria and found them to accurately predict postoperative complications, however, a new tumor size threshold of 38 mm was proposed based on the authors’ cohort [27]. Goh et al. found that the necessity of postoperative blood transfusion and length of hospitalization are well-differentiated by the Hasegawa score, the IMM and the SHH classification, with the SHH classification demonstrating the poorest but still accurate differentiation [15]. Interestingly, none of the scores incorporates the number of lesions resected as a difficulty evaluation criterion. However, the extent of liver resection and the location of lesions are ranked as major criteria for technical difficulty by the Ban score, IWATE criteria, the Hasegawa and IMM classification. Therefore, LLR for multiple lesions may not result in technical difficulty if they are small and easily accessible.

The study cohort was composed of patients with primary liver resection, secondary LLR or resection for recurrence were excluded. The rationale was to create a homogeneous cohort and to avoid introducing potential bias. In patients with secondary or recurrence lesions after previous liver surgery, technical difficulty can potentially be increased by varying levels of adhesions and impaired accessibility. Previous laparoscopic versus open approach, the extent of primary resection, the remaining liver function as well as the time interval between first and secondary surgery may have a considerable impact on technical difficulty and short- vs. long-term postoperative outcomes. The overall complexity of resections performed in the study cohort was very high, and for most scoring systems, the majority of LLR fell into the category of high or even expert difficulty. The respective difficulty scores rely on various distinct parameters to define technical difficulty. Several scores include the extent of resection and tumor size but none of them is exclusively relying on these parameters. Major additional aspects of technical difficulty as reflected by the respective scores are tumor location, proximity to major blood vessels, obesity and laboratory values such as platelet count. These parameters increased the difficulty score numbers in cases with minor resections for our cohort and led to a 18.9 − 53.2% rate of high difficulty cases despite 81% of cases being minor resections. Liver function and Child Pugh score are only incorporated in the IWATE score and are typically not considered a major factor of technical difficulty. Child Pugh scores were moderate. This is reflective of a high rate of patients that typically have no underlying liver disease (e.g. benign lesions, liver metastases or gallbladder cancer). Our results show differences to other surgical series validating the difficulty scores evaluated in this study. There are different potential explanations: Other series such as the study by Barron et al. also included robotic and hand-assisted procedures with different difficulty profiles. These studies further typically included a higher rate of HCC cases and therefore patients with underling liver disease. However, the rates of major postoperative morbidity was similarly low with less than 5% (13,14). Importantly, although postoperative major morbidity increased with the level of technical difficulty, the overall rates remain low at 0–5% in the context of the current literature ranging to up to 25% [24, 25, 28]. Postoperative mortality was very low at 1%. Optimizing preoperative preparation and patient work-up as well as intraoperative conditions are crucial for performing LLR, and complex LLR in particular. Preoperative work-up should include high-resolution imaging and thorough assessment of eligibility for a laparoscopic approach and lesion accessibility by the entire surgical team. An experienced laparoscopic liver surgery team should be involved for performing complex LLR, and a special focus should be given to optimal patient positioning and operating room ergonomics for long-duration procedures.

Besides aspects of technical difficulty related to anatomical location and extent or localization of liver lesions, other aspects remain important when selecting patients for LLR. Patients with advanced ASA scores or cardiopulmonary morbidities exacerbated by a prolonged pneumoperitoneum may not be good candidates for LLR. We recommend carefully considering patient-related factors when selecting patients for LLR. This study is limited in that it includes the experience of a single surgeon at a single institution.

While LLR is becoming more established at expert liver surgery centers in particular, robotic liver resections are increasing, and yielding promising results [4]. Important advantages are enhancement of dexterity and improved ergonomics for the surgeon [29]. While a recent multicenter propensity score based matched study showed superior results in terms of postoperative outcomes and conversion rates for robotic liver resection as compared to LLR [30], a recent randomized controlled trial deemed both techniques equivalent in outcomes [31]. It yet needs to be determined what the future role of robotic as compared to the currently more and more established laparoscopic liver surgery will be for complex procedures in particular.

This study has several limitations that need to be addressed. It is a single-center series of procedures performed by one experienced laparoscopic liver surgeon over a time period of more than 10 years. Therefore, the results are dependent on a single surgeon’s experience and cannot necessarily be reproduced depending on center setup and surgeon experience. LLR studies often necessarily cover a longer time period due to the extended learning curve of LLR and major LLR in particular. However, despite a growing number of structured training programs and an increasing use of laparoscopic techniques in liver surgery, the number of surgeons performing major LLR routinely at tertiary centers is still very limited. In this context, we think that evaluating difficulty scores for LLR in a single center series can still be valuable and instrumental for other laparoscopic liver surgeons. Further limitations of the study are its retrospective design and potential associated biases as well as a limited sample size, the low incidence of major complications and the evolution of surgical technique and instrument setup over time.

In conclusion, all technical difficulty scores performed well in differentiating intra- and postoperative major outcomes of LLR. The IWATE criteria performed best in stratifying all major outcome parameters. Major and minor laparoscopic hepatectomies can be performed with low intra- and postoperative morbidity at a high-volume specialized center. Careful patient selection and extensive LLR training are required to achieve low postoperative morbidity and mortality rates.

## Data Availability

No datasets were generated or analysed during the current study.
